# Small extracellular vesicles in metabolic remodeling of tumor cells: Cargos and translational application

**DOI:** 10.3389/fphar.2022.1009952

**Published:** 2022-12-16

**Authors:** Hao Yang, Jingyi Wang, Gang Huang

**Affiliations:** ^1^ Shanghai Key Laboratory of Molecular Imaging, Jiading District Central Hospital Affiliated Shanghai University of Medicine and Health Sciences, Shanghai, China; ^2^ Department of Nuclear Medicine, Shanghai Chest Hospital, Shanghai Jiao Tong University, Shanghai, China

**Keywords:** small extracellular vesicles, exosomes, warburg effect, tumor metabolism, liquid biopsy, glycolysis, drug development

## Abstract

Warburg effect is characterized by excessive consumption of glucose by the tumor cells under both aerobic and hypoxic conditions. This metabolic reprogramming allows the tumor cells to adapt to the unique microenvironment and proliferate rapidly, and also promotes tumor metastasis and therapy resistance. Metabolic reprogramming of tumor cells is driven by the aberrant expression and activity of metabolic enzymes, which results in the accumulation of oncometabolites, and the hyperactivation of intracellular growth signals. Recent studies suggest that tumor-associated metabolic remodeling also depends on intercellular communication within the tumor microenvironment (TME). Small extracellular vesicles (sEVs), also known as exosomes, are smaller than 200 nm in diameter and are formed by the fusion of multivesicular bodies with the plasma membrane. The sEVs are instrumental in transporting cargoes such as proteins, nucleic acids or metabolites between the tumor, stromal and immune cells of the TME, and are thus involved in reprogramming the glucose metabolism of recipient cells. In this review, we have summarized the biogenesis and functions of sEVs and metabolic cargos, and the mechanisms through they drive the Warburg effect. Furthermore, the potential applications of targeting sEV-mediated metabolic pathways in tumor liquid biopsy, imaging diagnosis and drug development have also been discussed.

## 1 Introduction

Tumor cells can rapidly utilize glucose through “aerobic glycolysis” and produce high levels of lactate—a phenomenon known as the Warburg effect. Hanahan and Weinberg characterized this metabolic reprogramming of tumor cells as one of the hallmarks of cancer, and a potential therapeutic target (Hanahan and Weinberg, 2011; Hanahan, 2022). The Warburg effect is primarily driven by the aberrant expression of key metabolic enzymes, production of oncometabolites, and overactivation of growth signals (Vander Heiden et al., 2009; Liberti and Locasale, 2016; Poff et al., 2019). The key enzymes of glycolysis, including hexokinase 2 (HK2), 6-phosphofructo-2-kinase/fructose-2,6-bisphosphatase-3 (PFKFB3) and pyruvate kinase M2 (PKM2), promote the conversion of glucose to lactate instead of pyruvate in the tumor cells, which in turn impairs mitochondrial oxidative phosphorylation (Minchenko et al., 2002; Yang et al., 2014; Xu and Herschman, 2019). Furthermore, glucose 6-phosphate dehydrogenase (G6PD) produces ribose and NADPH in the tumor cells through the pentose phosphate pathway (PPP) as a source of energy for tumor growth (Jiang et al., 2011; Deng et al., 2021). Therefore, understanding the complex mechanisms underlying glucose uptake and metabolism by tumors, and the role of the tumor microenvironment (TME), can improve diagnostic accuracy and aid in drug development.

According to the classification criteria of the International Society for Extracellular Vesicles (ISEV), small extracellular vesicles (sEVs) or exosomes measure less than 200 nm in diameter, and are formed by the fusion of multivesicular bodies with the plasma membrane. They are typically secreted by tumor cells and other cells in the TME into various body fluids (Théry et al., 2018; Armacki et al., 2020; Möller and Lobb, 2020; Wu et al., 2021). The sEVs contain a large number of functional proteins, nucleic acid fragments (including DNA, mRNA or non-coding RNA) and other biologically active substances from the parent cells, and thus mediate transport and information exchange between cells. Most tumor cells release higher amounts of sEVs compared to normal cells. The tumor-derived sEVs promote angiogenesis, induce chemoresistance and differentiation of stromal cells in the TME, and regulate immune responses and the pre-metastatic microenvironment (Li and Nabet, 2019; Mashouri et al., 2019; Yousefi et al., 2020). Studies show that the sEVs in TME can also regulate tumor metabolism by directly delivering metabolic enzymes, metabolites or other factors to the recipient cells (Zhao et al., 2016; Wang et al., 2020b). Furthermore, these metabolically reprogrammed sEVs also modulate glucose uptake by tumor cells and other TME components, including stromal cells, immune cells and fibroblasts (Zhao et al., 2016; Yan et al., 2018; Rai et al., 2019; Morrissey et al., 2021). In this review, we have summarized the mechanisms through which the sEVs mediate the crosstalk between tumor cells and the TME, and propose possible applications of targeting sEV-related metabolic pathways for precise diagnosis and effective treatment of tumors.

## 2 Sorting of metabolic cargoes into sEVs and secretion

**FIGURE 1:**
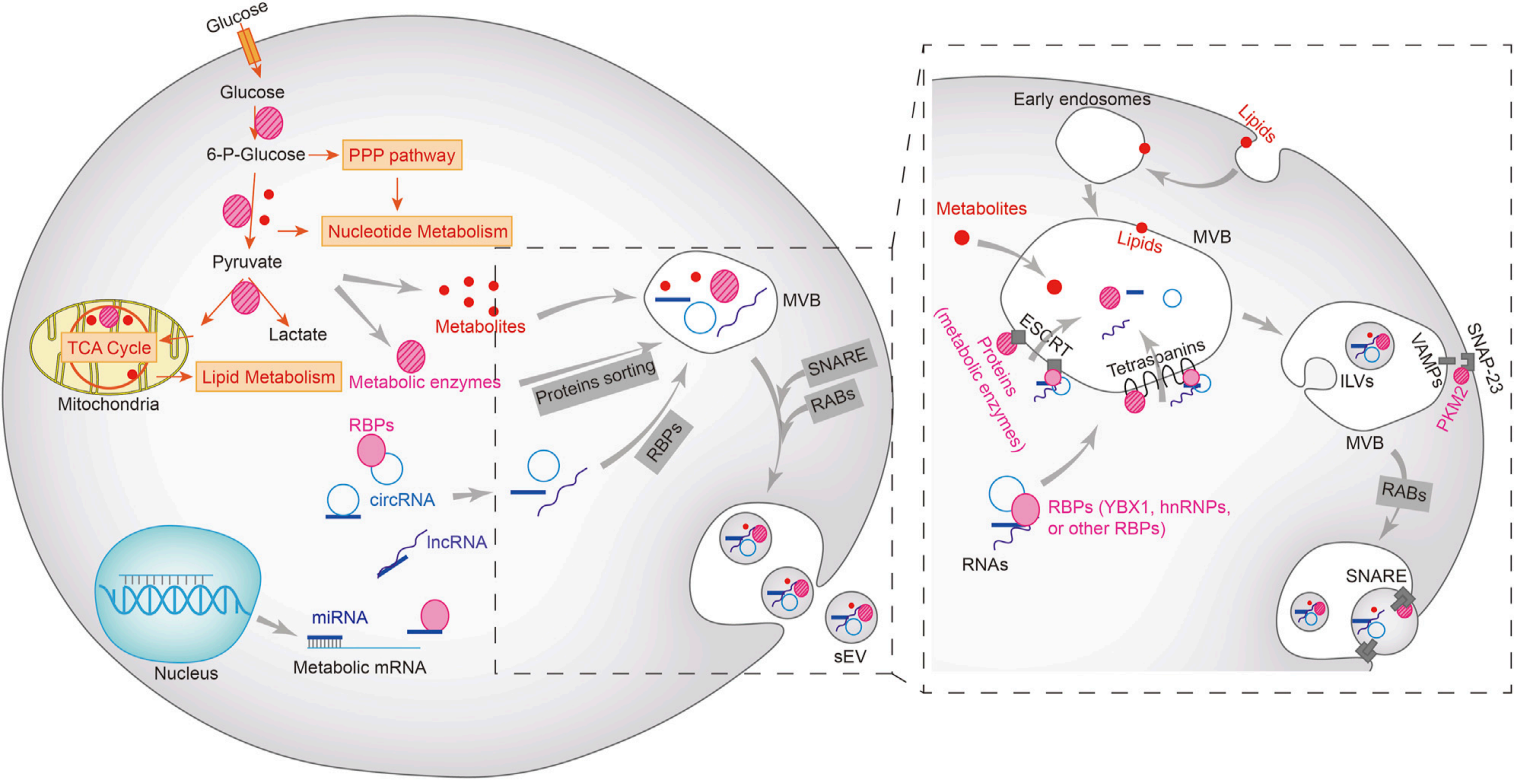
Metabolic cargoes in sEVs and their packaging, sorting and biogenesis. Metabolic enzymes and other regulatory proteins, metabolites, miRNAs, lncRNAs, and circRNAs are sorted into multivesicular bodies (MVBs) in different ways and subsequently excretes from the cells *via* the vesicle secretion pathways. Lipids (including phospholipids, sphingolipids, or triglycerides) on the plasma membrane enter MVBs *via* the early endosomes, but the sorting mechanism of other metabolites remains unclear. Proteins, including metabolic enzymes as well as RNA-bound RNA binding proteins (RBPs) enter the MVBs through ESCRT-dependent or independent mechanisms. The intraluminal vesicles (ILVs) in MVBs bind to the plasma membrane to form SNARE complex, which facilitates the extracellular release of sEV.

Tumor-specific sEVs can directly trigger the Warburg effect by transporting metabolites, enzymes, and the regulatory proteins and RNAs throughout the TME. The different intracellular components are loaded into sEVs through distinct sorting mechanisms (Figure 1). The biogenesis of sEVs is initiated following the inward budding of the membranes of multivesicular bodies (MVBs) to form intraluminal vesicles (ILVs). The mature MVBs fuse either with the plasma membrane and are expelled as sEVs, or with the lysosomes and are broken down (Anand et al., 2019). Rab GTPases act as the molecular switches that control ILV formation and transport within MVBs. The protein cargo is first sorted into the ILVs by tetraspanins and the endosomal sorting complex required for transport (ESCRT). VAMP2, VAMP3, VAMP7 and VAMP8 on the surface of MVBs form a SNARE complex with SNAP-23 on the plasma membrane, which releases the ILVs as sEVs (Clancy et al., 2019; Kumar et al., 2020; Zhang et al., 2021b). Pyruvate kinase M2 (PKM2) is co-sorted into the sEVs with the SNARE complex by phosphorylating SANP-23. In addition, the intra-vesicular PKM2 also promotes the exocytosis of sEVs from tumor cells (Wei et al., 2017; Fan et al., 2022). Lipids such as phospholipids and cholesterol are sorted into the sEVs by forming lipid rafts that are incorporated into sEV membranes (Zebrowska et al., 2019; Kumar and Deep, 2020). The sorting mechanism of other metabolites, including sugars, amino acids, nucleotides or vitamins, are not completely understood.

The mechanism underlying exosomal sorting of RNAs has been partially revealed (Liu et al., 2021). The RNA-binding Y-box protein I (YBX1) and Lupus La protein are required for loading miR-223 and miR-122 into sEVs, and is dependent on the high affinity of miRNAs for these RNA-binding proteins (Shurtleff et al., 2016; Temoche-Diaz et al., 2019). In fact, miRNAs that are sorted into sEVs possess similar motifs, and are enriched in the vesicles by specific RNA-binding proteins (Garcia-Martin et al., 2022b). For long RNAs such as mRNAs or lncRNAs, hnRNP A2B1 is responsible for binding to the corresponding motifs and sorting them into sEVs (O'Grady et al., 2022). The exact mechanism through which circRNAs are sorted into sEVs is still being explored, and studies suggest that it is likely accomplished through a miRNA binding-dependent manner or *via* direct interaction with RNA-binding proteins (Bao et al., 2016; Xu et al., 2020).

## 3 sEVs as drivers of Warburg effect

As already mentioned, the sEVs derived from tumor cells and TME components (fibroblasts, epithelial cells, macrophages, NK cells and other immune cells) reprogram glucose metabolism in tumor tissues by transporting proteins (metabolic enzymes, membrane proteins and other growth signaling proteins), nucleic acids (miRNAs, lncRNAs, circRNAs and mRNAs) and metabolites. The resulting dysregulation in metabolic pathways promote tumor progression, metastasis, and therapy resistance (Göran Ronquist, 2019; Zhang et al., 2021a). The contents of sEVs that regulate the Warburg effect are listed in Table 1.

**TABLE 1:** sEV cargoes and mechanisms involved in the regulation of the Warburg effect.

Cargo type	sEV cargos	Cells of origin	Target	Biological behavior or application	Reference
Protein	HK1/2/3	Hypoxic ovarian cancer	Glycolysis	Carboplatin resistance	Alharbi et al. (2021)
	PKM2	Hypoxic NSCLC, gastric cancer	Glycolysis	Cisplatin resistance	Wang et al. (2021), Dai et al. (2022), Wu et al. (2022), Zhou et al. (2022)
	ITGB4	TNBC	Glycolysis	Growth and metastasis	Sung et al. (2020)
	HMGB-1, PD-L1	Lung adenocarcinoma	Glycolysis, nitric oxide metabolism	Suppressive immunity of macrophages	Morrissey et al. (2021), Wei et al. (2021)
	LMP1	Nasopharyngeal carcinoma	Glycolysis, Oxidative phosphorylation	CAF formation, radioresistance	Aga et al. (2014), Wu et al. (2020)
	ALDOA, GAPDH, LDHA/B, PGK1, PKM1/2	HPV-driven oropharyngeal cancer	Glycolysis	Tumor diagnosis	Tang et al. (2021)
	IDH1	Colorectal cancer	NADPH metabolism	5FU resistance	Yang et al. (2021)
	ENO3, GALK1, ASNS, SCCPDH	Retinoblastoma	Glucose metabolism	Intravitreal tumor metastasis	Galardi et al. (2020)
	ANGPTL7	Ovarian cancer	Glucose and lipid metabolism	Angiogenesis	Parri et al. (2014)
	ABCG2	Pancreatic cancer	Glucose uptake	Gemcitabine resistance	Bhattacharya et al. (2014), Giovannetti et al. (2017)
miRNA	miR-122	Breast cancer	PKM2, Glycolysis	Brain/lung metastases	Fong et al. (2015), Li et al. (2019)
	miR-451	Gastric cancer	AMPK/mTOR	T cell immunity	Liu et al. (2018), Li et al. (2021)
	miR-543	Epithelial ovarian cancer	Proteoglycan pathway	Tumor proliferation	Zhang et al. (2022)
LncRNA	MALAT1	NSCLC	Glycolysis	Tumor proliferation	Zhang et al. (2017), Wang et al. (2020a)
	TUG1	CAF	Glycolysis	Lung metastases of liver cancer	Lu et al. (2022)
	HISLA	Tumor-associated macrophages	Glycolysis	HIF-1α	Chen et al. (2019)
CircRNA	circ_0005963	Colorectal cancer	PKM2, Glycolysis	Oxaliplatin resistance	Wang et al. (2020c)
	circ-RNF121	Colorectal cancer	Glycolysis	Invasion and metastasis	Jiang et al. (2021)
	circ_0008928	NSCLC	HK2, Glycolysis	Cisplatin resistance	Shi et al. (2021)
	circ_0072083	Glioma cells	Glycolysis	TMZ resistance	Ding et al. (2021)
	circCCT3	Liver cancer related CAF	HK2, Glycolysis	Tumor proliferation	Lv et al. (2020)
	circARHGAP10	NSCLC	Glycolysis	Tumor proliferation	Fang et al. (2022)
Metabolites	Lactate, acetate, stearic acid, palmitic acid, amino acid	Prostate and pancreatic cancer related -CAF	Central carbon metabolism	Kras-dependent tumor growth	Zhao et al. (2016)
	Phosphatidylcholine	Mouse colon cancer cells	Lipid metabolisim	Insulin signaling resistance	Kumar et al. (2021)
	Linoleic acid	Lung adenocarcinoma	Glycolysis, nitric oxide metabolism	Suppressive immunity of macrophages	Morrissey et al. (2021)

### 3.1 Metabolic enzymes in sEV

The hypoxic environment within solid tumors is a major inducer of Warburg Effect. Ovarian cancer cell-derived sEVs load higher levels of hexokinase (HK), UDP-glucuronosyltransferase, 6-phosphogluconolactonase and CTP synthase 1, which are the key enzymes in glucose, phospholipid and nucleic acid metabolism, when exposed to hypoxic conditions (Alharbi et al., 2021). These sEVs loaded with metabolic enzymes are critical for the development of platinum resistance in ovarian cancer. In a previous study, we showed that cisplatin-resistant non-small cell lung cancer (NSCLC) cells transmitted drug resistance to sensitive cells by secreting PKM2-loaded exosomes in response to hypoxic stimulation (Wang et al., 2021). Thus, inhibiting glucose metabolism could potentially reduce sEV-mediated chemoresistance in cancer cells. For example, pancreatic cancer cells with autophagy blockade exhibited low glucose uptake and expressed low levels of ATP-binding cassette sub-family G member 2 (ABCG2) in the secreted exosomes, which restored gemcitabine sensitivity (Bhattacharya et al., 2014). Although oxidative phosphorylation is attenuated in tumor cells, some enzymes of the Krebs cycle are also released within sEVs. We previously observed that exosomal release of cytoplasmic IDH1 promoted 5FU resistance in colorectal cancer cells (Yang et al., 2021). In addition to chemoresistance, exosomal metabolic enzymes can also enhance other malignant features such as invasion, metastasis, angiogenesis and immune escape. For example, exosomal angiopoietin-like protein 7 (ANGPTL7) secreted by the human ovarian cancer cells can promote tumor angiogenesis by regulating glucose and lipid metabolism, and oxidative stress (Parri et al., 2014). Several studies in recent years have established the presence of metabolic enzymes in sEVs and their impact on oncogenic signaling networks. Retinoblastoma-derived exosomes are enriched in multiple metabolic enzymes, including enolase 3 (ENO3), galactokinase-1 (GALK1), asparagine synthase (ASNS) and saccharopine dehydrogenase (SCCPDH) (Galardi et al., 2020). Taken together, transmission of metabolic enzymes *via* sEVs affects tumor progression through metabolic or non-metabolic signaling pathways.

### 3.2 Metabolites in sEV

Tumor or TME-derived sEVs can modulate tumor progression by transporting specific metabolites to distant tissues, immune cells, *etc.* For instance, colon cancer cells expressing CD63, CD9 and A33 are known to deliver exosomal phosphatidylcholine to liver tissues *via* the portal vein (Kumar et al., 2021). Furthermore, lung adenocarcinoma-derived exosomes are enriched in linoleic acid (LA) compared to those derived from normal lung epithelial cells, and promote tumor metastasis by reprogramming macrophage glycolysis and PD-L1-dependent immunosuppression (Morrissey et al., 2021). Metabolomics have been used to identify the species, content and signaling pathways of metabolites in sEVs and other vesicles. Metabolite analysis of CAF-derived exosomes by gas chromatography-mass spectrometer (GC-MS) and ultra-high performance liquid chromatography (UPLC) revealed high levels of intermediates produced during glucose metabolism (such as citric acid and pyruvate), lipids (stearic and palmitic) and most amino acids (Zhao et al., 2016). However, the exact functions of these exosomal metabolites in tumor progression remains to be explored.

### 3.3 Nucleic acids regulate tumor metabolism *via* sEV

Exosomal nucleic acids, especially non-coding RNAs, are established diagnostic biomarkers of various tumors. Non-coding RNAs can promote or impede the Warburg effect by targeting specific metabolic enzymes or regulatory genes. For example, colorectal cancer cell-derived exosomal circ-RNF121 enhanced glycolysis and reduced ATP/ADP production in the tumor cells (Jiang et al., 2021). Furthermore, circ-0008928 is highly expressed in NSCLC cell-derived sEVs, and confers cisplatin resistance by acting as a “sponge” for miR-488, which enhances HK2 activity and glycolysis (Shi et al., 2021). NSCLC cells also secrete the lncRNA MALAT1 *via* sEVs, which is known to promote lactate dehydrogenase A (LDHA) expression and glycolysis (Wang et al., 2020a). Oxaliplatin-resistant colorectal cancer cells can transmit chemoresistance to the sensitive cells through exosomal circ-0005963, which sponges miR-122 and upregulates PKM2 (Wang et al., 2020c). Exosomal RNA-mediated decrease in glucose uptake in the normal tissues also contributes to tumor development. For instance, breast cancer cell-derived exosomal miR-122 drives brain/lung metastasis by downregulating PKM2 and inhibiting glucose uptake in distant non-tumor tissues (Fong et al., 2015). In addition to tumor cells, the stromal and immune cells of the TME also secrete sEVs that reprogram tumor metabolism. There is evidence that exosomal lncRNA TUG1 secreted by cancer-associated fibroblasts (CAFs) in HCC tissues increases LDH activity and lactate production in the tumor cells (Lu et al., 2022).

Interestingly, the metabolic reprograming induced by exosomal components exert a positive feedback by increasing cargo loading into the sEVs. For example, exosomal circ_0072083 derived from temozolomide (TMZ)-resistant glioma cells activates glycolysis and increases the levels of glucose transporter 1 (GLUT1), LDHA and PKM2, which further induce the expression of the circRNA and amplify TMZ resistance (Ding et al., 2021). Furthermore, the dissemination of sEVs in the TME also depends on the extracellular glucose status. Under energy-deficient low-glucose conditions, gastric cancer-derived exosomal miR-451 is more inclined to polarize T cells to the immunosuppressive Th17 cells, which leads to poor prognosis (Liu et al., 2018). In fact, tumor-derived sEVs can also trigger metabolic disorders, as exemplified by the interaction of pancreatic cancer and diabetes (Sah et al., 2019). Pancreatic cancer cell-derived exosomes delivered miR-19a to islet β cells and disrupted normal insulin secretion (Pang et al., 2021; Su et al., 2022), and reprogramed enteroendocrine cell function *via* multiple exosomal miRNAs (Zhang et al., 2018). Thus, TME-derived sEV RNA reprograms tumor glucose metabolism and creates favorable conditions for tumor cell growth and metastasis, while the sEV RNA cargo secreted by metabolically abnormal tumor cells also enhances the malignant potential.

### 3.4 Resident proteins in sEV

The sEVs derived from different tissues may express similar marker proteins, including CD9, CD63, CD81 or TSG101, which are used for their identification. However, most of these ubiquitous resident proteins of sEVs, most of which are membrane proteins, show differential expression across cell types during proteomics examination (Kowal et al., 2016; Rai et al., 2021; Garcia-Martin et al., 2022a). Some resident exosomal proteins are transferred to recipient cells, wherein they reprogram glucose metabolism. For example, nasopharyngeal carcinoma cell-derived sEVs can transform normal fibroblasts to a tumor-promoting phenotype by delivering latent membrane protein 1 (LMP1), which enhances glycolysis and inhibits mitochondrial oxidative phosphorylation *via* activation of the NF-κB pathway (Wu et al., 2020). Integrin beta 4 (ITGB4) is highly expressed on triple-negative breast cancer (TNBC) cells and their derived exosomes, and promotes glycolysis in CAFs through mitophagy (Sung et al., 2020). High mobility group box-1 (HMGB-1) is highly expressed in lung adenocarcinoma exosomes and promotes glycolysis in the tumor macrophages by upregulating PD-L1 expression (Morrissey et al., 2021). The 78 kDa glucose-regulated protein (GRP78) is translocated from the endoplasmic reticulum to the plasma membrane, and released *via* exosomes (Li et al., 2016; Gonzalez-Gronow et al., 2021). High expression of exosomal GRP78 in colorectal and breast tumors is associated with increased glycolysis and metastasis (Zheng et al., 2019; Li et al., 2022). Therefore, proteins that are differentially expressed in the tumor-derived exosomes relative to that secreted by normal cells should be considered as potential as biomarkers. Overall, tumor cell-derived sEV-resident proteins act as “villain messengers” by promoting malignant behaviors such as invasion, metastasis and immunosuppression through metabolic reprograming.

## 4 Application of targeting metabolic sEVs

Given their unique protein profiles, sEVs are promising biomarkers for early, non-invasive tumor detection, and for monitoring therapy (Liang et al., 2021). Tumor-derived exosomal protein or RNA cargoes can be easily detected in liquid biopsies for rapid diagnostic and prognostic evaluation. Furthermore, inhibitors of sEV secretion or the loaded cargos potential drug candidates for tumor therapy. In addition, *in vivo* imaging of sEVs can be used as a surrogate for tracking tumor progression and therapeutic efficacy.

### 4.1 Metabolic biomarkers for liquid biopsy

Proteomic mass spectrometry analysis of salivary exosomes from human papillomavirus (HPV)-driven oropharyngeal cancer patients revealed significantly elevated aldolase, glyceraldehyde dehydrogenase-3-phosphate dehydrogenase (GAPDH), LDHA/B, phosphoglycerate kinase 1 (PGK1) and PKM1/2 (Tang et al., 2021). Compared to healthy volunteers, serum-derived exosomal circARHGAP10 was upregulated in NSCLC patients, and associated with increased the expression of GLUT1 and LDH, which are drivers of NSCLC progression (Fang et al., 2022). Furthermore, presence of serum-derived exosomes with low levels of miR-543 in patients with epithelial ovarian cancer indicates favorable prognosis since downregulation of this miRNA inhibits glucose uptake by tumor cells (Zhang et al., 2022). Cancer stem cells (CSCs) drive tumor initiation, drug resistance and metastasis, and are the primary cause of tumor recurrence. Metabolomics analysis of CSCs-enriched primary melanoma tissues and serum-derived exosomes from melanoma patients revealed aberrant expression of multiple metabolites (Palacios-Ferrer et al., 2021). Thus, exosomal metabolites in liquid biopsies are ideal markers of tumor diagnosis, therapeutic efficacy and prognosis.

### 4.2 Therapeutic agents targeting metabolic regulators

Some small molecule drugs and natural compounds can inhibit tumor-associated sEV secretion or metabolic pathways, and are thus potential candidates for tumor therapy. The AMPK inhibitor compound C and the c-Jun inhibitor SP600125 blocked the delivery of exosomes from the TNBC cells into CAFs, which inhibited glycolysis and tumor cell proliferation (Sung et al., 2020). The sEV-mediated immunosuppression is partly attributed to the metabolic reprogramming of TME, and drugs targeting sEV-related tumor immunity are suitable for tumor therapy, such as immune checkpoint inhibitors (Marshall and Djamgoz, 2018; Bader et al., 2020; Yang et al., 2020). As already described, tumor cell-derived exosomes increased glucose uptake and PD-L1 expression in CAFs in an NF-kB-dependent manner, which was inhibited by the glycolysis inhibitor 2-deoxyglucose. Furthermore, tumor sEVs also inhibited oxidative phosphorylation in tumor-associated macrophages by upregulating NOS2 expression, and administration of the NOS2 inhibitor S-ethylisothiourea hydrobromide restored oxygen utilization (Kuruppu et al., 2014; Morrissey et al., 2021). In addition, coptisine disrupted the secretion of exosomal circCCT3 from CAFs and reduced the expression of HK2 in liver cancer cells (Lv et al., 2020), which has potential therapeutic value. The natural compound oleanolic acid reduced the secretion of exosomes from TMZ-resistant glioma cells, and shikonin inhibited resistant cells-derived exosomal circ_0072083 levels and the Warburg effect (Ding et al., 2021). These compounds have potential sensitizing effects on TMZ treatment of glioma. Therapeutic agents targeting sEV-related communications have also been shown to multidrug resistance -associated protein (MRP) -mediated resistance. The calmodulin-dependent protein kinase inhibitor KN-93 was used to reverse MSC sEV-mediated 5-FU resistance in gastric cancer (Ji et al., 2015). Mechanistically, these sEVs enhanced the expression of MRP and P-glycoprotein by regulating miR-301b and ERK kinase pathways (Ji et al., 2015; Zhu et al., 2022), which are inducers of glucose metabolism and malignant tumorigenesis (Asl et al., 2021; Jandova and Wondrak, 2021). However, the limitation lies in the lack of the effect of sEV from other TME components (other than MSC) on MRP and chemotherapy resistance. More studies are needed to explore the mechanism of chemotherapy resistance of metabolic sEV in order to develop therapeutic agents with relevant targets.

### 4.3 Metabolic imaging of sEVs in nuclear medicine

Due to the sentinel role of exosomes in early TME shaping and formation of the pre-metastatic niche, *in vivo* sEV imaging can be helpful for early tumor monitoring. At present, sEV imaging mainly focuses on tracking the fluorescently-labeled sEVs. For example, plasma membrane-specific fluorescent dyes such as DIR and PKH26 are commonly used to label exosomes and track them at the cellular level (Pužar Dominkuš et al., 2018; Sun et al., 2019; Kumar et al., 2021). Nuclear imaging, including ^18^F-deoxyglucose (FDG)-positron emission tomography/computed tomography (PET/CT), is currently the main diagnostic method for evaluating tumor glucose uptake and metabolic activity, and offers the possibility for isotopic labeling and imaging of exosomes. Engineered exosomes labeled with radioisotopes (including ^99m^Tc, ^111^I, ^125^I, ^131^I, ^64^Cu, ^68^Ga) have achieved non-invasive PET or SPECT detection in animal models, and show promise for clinical translation (Morishita et al., 2015; Gangadaran et al., 2018; Rashid et al., 2019; Jung et al., 2020; Lázaro-Ibáñez et al., 2021).

Radio-labeled exosomes have excellent intra-tumoral homing ability and biosafety (Shi et al., 2019). FDG-PET imaging has been used to assess the function of exosomes in Alzheimer’s disease development since glucose metabolism in the affected tissues correlates with the secretion of plasma-specific exosomes (Coumans et al., 2017; Chanteloup et al., 2019). In addition, FDG-PET imaging has also demonstrated the activation of glucose metabolism in breast tumor cells through EVs uptake (Kang et al., 2021). Furthermore, the enriched miRNA levels in plasma EVs from Hodgkin lymphoma patients matched the FDG PET imaging results, suggesting that EVs can be used for metabolic tracing in tumor patients (van Eijndhoven et al., 2016). In an *in vitro* study, ^13^C isotope-labeled glucose was used to evaluate the effect of CAF-derived exosomes on the metabolites of glycolysis (Zhao et al., 2016). Although isotopic tracing of exosomes can be used for tumor imaging, very few studies have reported direct radiolabeling of metabolites in EVs for *in vivo* imaging.

## 5 Conclusion and perspectives

**FIGURE 2:**
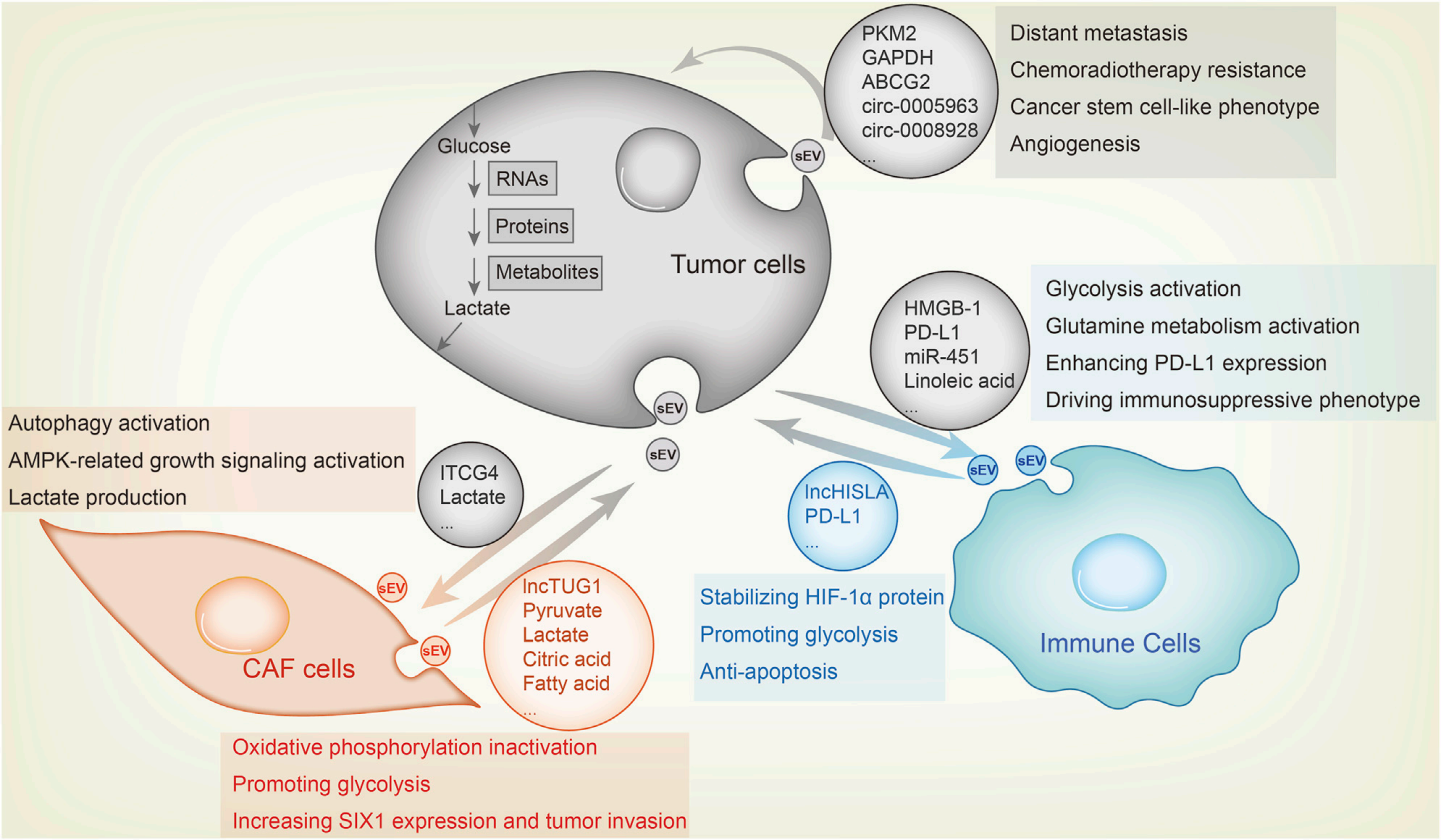
Metabolic sEV exchange between tumor cells and other components in the TME. The uptake of tumor-derived sEVs by CAFs or immune cells facilitates tumor growth, whereas tumor cell fusion of metabolic sEVs in TME leads to its malignant transformation. For example, CAFs-derived sEV lncRNA TUG1 delivers to liver cancer cells, increasing SIX1 expression and promoting glycolysis and tumor invasion. CAFs-derived sEVs inhibit oxidative phosphorylation and activates glycolysis by delivering lactate, pyruvate, citric acid, fatty acids and amino acids to pancreatic cancer cells. As feedback, breast cancer cells sEVs carry ITGB4 proteins that can be transmitted to CAFs, inducing autophagy, AMPK activation and lactate production in CAFs, which contribute to tumor invasion. As for the effect of sEV on immune cells, lung cancer-derived sEV HMGB1, PD-L1 and linoleic acid enhance glycolysis, glutamine metabolism and PD-L1 expression in macrophages and T cells through NF-κB signaling, endowing these immune cells with an immunosuppressive phenotype. MiR-451 in sEV of gastric cancer transmits to T cells and enhances their infiltration, causing them to differentiate to Th17 cells and forming malignant transformation of tumor microenvironment. Macrophages also secrete sEVs, which enhance aerobic glycolysis and anti-apoptosis of breast cancer cells by delivering lncRNA HISLA and stabilizing HIF-1α protein. Among tumor cells, highly metastatic cells, chemotherapy or radiotherapy resistant cells and cancer stem cell-like cells transfer sEV to sensitive cells to confer corresponding malignant phenotype.

The Warburg effect is manifested as overactive glycolysis and aberrant activity of metabolic enzymes, and is instrumental for the rapid proliferation, invasiveness, drug resistance and immune escape of tumor cells. Extracellular vesicles (EVs) mediate intercellular communication in the TME, and the sEVs or exosomes have been studied the most. In this review, we outlined the mechanisms through which proteins, RNAs and metabolites are sorted into the sEVs within cells, as well as the pathways regulated by the exosomal cargo to remodel tumor metabolism and promote tumor progression (Figure 2). Furthermore, we have also discussed the diagnostic and therapeutic potential of tumor-derived sEVs.

Analysis of exosomal protein mass spectrometry and sequencing data form EV databases such as ExoCarta, exoRBase or Vesiclepedia have also indicated that the resident metabolic enzymes or RNAs may be involved in regulating tumor metabolism (Kalra et al., 2012; Keerthikumar et al., 2016; Lai et al., 2021). Therefore, more sEV cargoes and functions may be discovered in the future, and provide new insights into tumor metabolic remodeling. Although some carbohydrate-metabolizing enzymes (such as HK2, ENO3 and PKM2) delivered *via* sEVs are known to remodel the TME, given the opposing functions of glycolytic and mitochondrial metabolic enzymes in the Warburg effect, it remains to be ascertained whether these enzymes exert different functions when sorted into sEVs and in the recipient cells. In addition, since tetraspanins may potentially regulate nutrient metabolism in tumor cells, their use as a biomarker of sEV protein cargo also needs to be considered (Wang et al., 2019; Najy et al., 2021).

The diagnostic, therapeutic and imaging applications of tumor-derived sEVs has gained considerable attention in recent years. Tumor patient-derived sEVs containing metabolic enzymes or miRNAs are diagnostic biomarkers, which opens up the possibility of developing rapid sEV isolation and microfluidic chip analysis of the cargo. ^18^F-deoxyglucose (FDG) is routinely used as a tracer for the radiometabolic imaging of tumors during diagnosis and treatment evaluation (Jacobson and Chen, 2013; Yang et al., 2017; Sheikhbahaei et al., 2020). However, the clinical utility is somewhat limited. For example, FDG PET/CT is prone to inflammatory interference in cervical cancer, resulting in low diagnostic specificity (Dejanovic et al., 2021). In addition, tumors such as hepatocellular carcinoma (HCC) have a lower capacity for FDG uptake (Li et al., 2018). Therefore, isotope probes targeting tumor-specific sEVs offer more possibilities for the diagnosis of heterogeneous tumors than FDG. Although sEVs can be labeled with fluorophores or isotopes for *in vivo* tumor imaging, the metabolic components of sEVs cannot be directly labeled as yet. The development of metabolic enzyme-targeted probes or radionuclide-labeled metabolites will be a significant progress in tumor imaging and clinical decision-making. Finally, more therapeutic or engineered agents targeting the metabolic cargo in sEVs need to be developed as drug delivery systems. In conclusion, targeting metabolic sEVs will play an important role in tumor diagnosis and therapy.
